# Understanding Jordanian Midwives’ Experiences of Providing Care during the COVID-19 Pandemic Crisis: A Phenomenological Study

**DOI:** 10.30476/ijcbnm.2021.88793.1545

**Published:** 2021-07

**Authors:** Karimeh Alnuaimi

**Affiliations:** Department of Maternal and Child Health Nursing, Faculty of Nursing, Jordan University of Science and Technology, Irbid, Jordan

**Keywords:** Coronavirus, Covid-19, Experience, Jordanian, Midwives

## Abstract

**Background:**

Midwives are at high-risk during the coronavirus disease (COVID-19) pandemic outbreak. Risks include virus exposures, extra working hours, psychological stressors, fatigue, work burnout, stigma, and physical and psychological violence. However, attention is needed to prepare the midwives during this crisis and help them overcome their challenges. The current study aims to explore Jordanian midwives’ experiences of providing health care during the COVID-19 pandemic crisis

**Methods:**

A descriptive phenomenological qualitative study was used. Semi-structured interviews using telephone calls were conducted to collect data from April to May 2020. Each interview lasted for 30-45 minutes. Purposive and snowball sampling strategies were used to recruit 20 midwives from two hospitals. Colaizzi’s method was used to analyze the data manually

**Results:**

Four main themes and eight subthemes were obtained. The main themes included: “Living in turmoil”; “Communication dilemma”; “Positive experience over time”, and “Seeking support”.

**Conclusion:**

Jordanian midwives experienced a stressful and interruptive routine life while providing care to their clients during the COVID-19 pandemic. This study recommends more attention towards the psychological state and factors that might affect the midwives during this crisis. The management and administrative teams should do their best to reduce the work pressure and arrange holidays appropriately. Policymakers and the government should provide the midwives with a safe work environment, good information resources, and financial rewards.

## INTRODUCTION

In December 2019, coronavirus disease, also known as COVID-19, emerged as an outbreak and caused serious complications among the Chinese people. However, the severity of this disease varies as some infected people do not experience any symptoms and others need critical care and treatment. ^1^ In Jordan like many countries in the world, the World Health Organization (WHO) confirmed that the first infected case was on the 2nd of March 2020 in the city of Irbid. ^2^ Health care professionals (HCPs) are considered a high-risk group who were expected to be affected during the epidemic and pandemic outbreak as they work in the frontline with patients. ^3^ Previous studies on COVID-19 outbreaks highlighted the negative impact on nurses such as physical complaints, fatigue, burnout, stigma, and emotional and psychological distress. ^4 - 6^ To decrease these negative outcomes, WHO is working constantly and has highlighted five crucial methods including offering accurate information to fight the outbreak, assisting the countries in responding, preparing by helping in building their capacity in collaboration with other agencies, providing the medical equipment and safety stuff for frontline nurses and midwives, and conducting research studies and training of HCPs. ^3^

The healthcare system in Jordan includes public and private sectors that offer services to Jordanians and other people from neighboring countries. Midwives provide different services for pregnant women and children in hospitals and health care centers including antenatal, childbirth, postnatal, family planning, newborn and child health, breast feeding, and immunization. Primarily, antenatal and postnatal care is provided in primary health care centers, while labor and birth take place at hospitals. ^7 , 8^ Jordan has a high shortage of midwives; however, the Ministry of Health started to work with the minimum number of HCPs including midwives to decrease the spread of the infection. ^2^ Despite all interruptions during the COVID-19 pandemic, studies about midwives’ experiences during this period have not been conducted in Jordan and even such studies are scarce in the world. The focus of this limited number of studies have been on the psychological and mental status of HCPs working with patients with COVID-19 and predominantly examined the viewpoints of nurses only, rarely including midwives, and mainly using quantitative approaches. ^4 - 6^ In the present study, the researcher used qualitative approach because advocates note the strength of qualitative methods in delivering a deeper understanding of the world of the midwives from their own perspective. ^9^ Descriptive phenomenology focuses on personal experiences and meanings as participants speak through language using such an approach. However, they actually present their psychological world with what they say and how they perceive their living conditions. ^10^ A deeper understanding of these unknown aspects reveals the actual needs of midwives to facilitate the planning of good strategies and implement them to improve the quality of care. Therefore, the aim of the current study was to explore the midwives’ experiences and perceptions during the COVID-19 outbreak using descriptive phenomenological approach.

## MATERIALS AND METHODS

A descriptive phenomenological qualitative design was used. Descriptive phenomenology is concerned with revealing the “essence” or “essential structure” of any phenomenon under investigation. Phenomenology is also the lived experience of humans from the world of their everyday lives. These experiences tell them what is real and true in life and gives meaning to their perception of a particular phenomenon which can be influenced by internal and external factors. ^10^ The chosen methodology in the current study is not intended to provide generalized results, but to understand the experience of midwives who provide maternal care during the COVID-19 outbreak and explore their real needs and challenges.

Twenty midwives participated in this study. Purposive sampling was used to recruit 13 midwives first. Then, the snowball sampling method was used to reach data saturation. The inclusion criteria were all Jordanian midwives who are working at maternity wards in the selected settings during the period of the Corona pandemic for at least two weeks and had more than two years of experience. Any midwife who took unpaid leave or did not work during the crisis period was excluded. The study participants were recruited from Princess Badea Training Hospital and Princess Raya Hospital in the city of Irbid, the third largest city in Jordan.

After receiving the consent to participate in the study, a suitable time for the interview was confirmed with each participant. Then, each participant chose a suitable time for her to undertake the interview, either from her home or from her work during the break time. Each participant was asked to verbally consent to use a digital recorder. Codes were used so that the real names of the participants would not be identified and to maintain anonymity in the reporting of direct quotes to support the study themes. For example, participant 1 refers to the first midwife who was interviewed.

Semi-structured interviews using telephone calls were conducted during the period from April to May 2020. In the current situation of curfew in Jordan, telephone calls were the most appropriate method for data collection. Many methodological studies found several benefits of telephone interviews including increased interviewer and interviewee’s safety, reduced costs, and greater ﬂexibility for scheduling. ^11 , 12^ However, shorter time than face-to-face interview and missing body language were documented as disadvantages of telephone interviews. ^13^ Midwives in this study, however, were interested in the topic and talked for at least 30 minutes. Further, the participants’ tone of voice was recorded and helped to understand the midwives’ experiences. 

All interviews were conducted by the author in the Arabic language. Data collection continued until saturation was reached (no new categories and themes were revealed). The main open-ended question was: “How do you describe your experience in providing care to women during this crisis (Corona pandemic)?”

Examples of further questions included: 

1. Please explain your experience if you have any challenges in this period other than what you experienced before the crisis? 

2. What kind of support and special facilities you receive to enhance your care in this period?

3. Please, from your experience, what are your requests or recommendations to improve your ability to deal with this crisis? 

To encourage the midwives to speak more about their experiences and gain an in-depth understanding, probing questions such as “Can you tell me more about your experience,” or “Can you explain that further?” were asked. All participants’ potentially identifying information was deleted. During the interviews, the participants were placed on the telephone speaker so that the interviews could be digitally recorded. All interviews were conducted and transcribed verbatim by the author. Each interview lasted for 30-45 minutes.

Colaizzi’s (1978) process for phenomenological data analysis with seven steps was used and occurred simultaneously with data collection. ^14^ Each transcript was read and re-read several times. Significant statements that relate to the midwives’ experiences were extracted for each transcript. All extracted statements were documented on a separate sheet noting their page and line numbers. Then, all meanings were formulated from these significant statements. Categories, clusters of themes, and themes were formulated. The findings were integrated into an in-depth description of midwives’ experiences and checked by an expert researcher in qualitative research to ensure richness and completeness of the findings. Then, the fundamental structure of the phenomenon was described. Regarding the process of translation, all results were double checked by a bilingual translator who is competent in both Arabic and English. An external auditor who reviewed the whole process of the study and performed an additional checking for the coding process and analysis was used. ^15^

Trustworthiness of the study was ensured by the credibility, transferability, and conformability of the findings that was maintained throughout the study using methods of validation of the findings of qualitative studies. ^16^ To assure credibility, member checking approach was used. The researcher discussed the findings with five respondents to ensure that the data and the interpretations of the findings reflected their experiences using phone calls. All midwives were happy and satisfied with the results. To assure transferability (or fittingness), we established “thick description”. Detailed description of the research process, settings and data was collected with a rich mix of participant’s quotations. “Bracketing” was the process used to assure conformability. Bracketing eradicates any bias inherent in the researcher beliefs and attitudes and allowing phenomena to speak about itself. ^15 , 17^ Finally, cross-checking of the whole analysis process was done by a qualitative research expert. 

The Institutional Review Board at Jordan University of Science and Technology in Irbid approved the study (Ref: 111/132/2020). Then, all midwives were informed about the study through the manager midwives in the selected hospitals. Next, the participants were asked for their contact numbers so that all details about the study would be sent to all participants via WhatsApp or text message. All participants gave their consent via WhatsApp and were asked again for their verbal consent at the start of the interview. All midwives were informed that their participation is voluntary and that they can withdraw at any time without consequences. They were assured that information given would be anonymous and confidential. Midwives were also informed that there were no risks in participating.

## RESULTS

In this study, 20 midwives with a mean age of 37 years old (range of 32-46 years) were interviewed. Their educational level varied from diploma to Master’s degree. The average working experience for all participants was 15.2 years, with a range of 10-25 years. The demographic characteristics of the twenty midwives are presented in Table 1. 

**Table1:** Demographic characteristics of the participants

Participant	Age	Education level	Experience (years)	Working department	Marital status	Number of children
1	32	Diploma	10	Labour	Single	0
2	37	Diploma	15	Labour	Married	3
3	34	Master	11	Labour	Married	1
4	40	Diploma	17	Antenatal	Married	4
5	39	Diploma	18	Labour	Married	2
6	32	Bachelor	10	Labour	Married	1
7	46	Diploma	25	Administration	Married	4
8	39	Diploma	14	Labour	Divorced	2
9	37	Diploma	16	Emergency	Married	2
10	36	Diploma	15	Labour	Marred	2
11	34	Bachelor	12	Labour	Married	4
12	39	Master	18	Administration	Single	0
13	36	Diploma	14	Labour	Married	3
14	38	Diploma	16	Emergency	Married	2
15	40	Diploma	19	Postnatal	Married	4
16	35	Master	11	Labour	Married	2
17	33	Bachelor	10	Labour	Single	0
18	40	Diploma	17	Antenatal	Married	3
19	37	Diploma	15	Labour	Married	4
20	43	Diploma	22	Postnatal	Married	5

The interviews explored how the participants perceived their experiences in providing care during the COVID-19 pandemic crisis. Also, they revealed the participants’ feelings, emotions, and thoughts about themselves, clients, and colleagues during this crisis. Finally, the findings formulated the midwives’ needs in more depth. Four themes emerged: “Living in turmoil”, “Communication dilemma”, “Positive experience over time”, and “Seeking support”. These themes were derived from eight sub-themes, as shown in Table 2.

**Table2:** The meaning units, sub-themes, and main themes

Meaning units	Subthemes	Themes
This virus scared me because we do not know the affected person	Fear from contamination with the virus	Living in a turmoil
I feel myself as a virus resource
I suppose myself affected all the time
I do not know if I affected by this virus
I have been scared and stressed at home more than work	Fear on Beloved Ones and clients
I afraid from unknown (voice with fear)	
I feel guilty if I kiss my children	
I afraid on my little girl (crying)	
My parents are old and I afraid to transfer the virus to them (sad voice)	
We are responsible of mothers and babies…my fear to harm them	
Too much work	Being physically tired from high workload
Client number reached 140 on the shift
Triple our work before
We getting tired
No time to go toilet or pray
Exhausted
Getting warm	Being uncomfortable from extra precautions
Difficult to breath with mask	
Sweating	
Hand dryness	
Cough	
Allergy	
Inhalation same breath	
Difficulty to perform procedure	
I’m not like before with patient	Midwife- client communication	Communication dilemma
I minimize the physical contact		
I call the doctor more than before		
Woman’s right to receive care as before		
No change in my communication		
I provide the best care as usual		
No kisses or hugs with colleagues	Midwife-colleague communication	
No coffee break as before		
I cannot avoid my close friend		
I’m still drink coffee as before		
I accept the way of work	Becoming less overwhelmed	Positive experience over time
My Fear was in the first week more than now
The situation now is better
It becomes norm now
Using protections is norm now
I get more information	Knowledge enhancement
I had a workshop on coronavirus disease	
I received more information	
I know about precautions but not about mother and her baby health	Need to access information resources	Seeking support
Coronavirus and pregnancy		
We need electronic journals access		
I do not know how to answer the woman if she asks me about		
I need more information		
We need valid resources		
Need more colleagues on the shift	Need to improve the work environment	
We are tired we need help		
The number of midwives is not enough		
Arrange the holiday but we need more midwives on the shift		
All other professionals stay at home and receive their salary		
We are the first line care providers		
The Ministry of Health should appreciate us		
Managers should help us to gain incentives		

### 1. Living in Turmoil

#### 1.a. Fear from Contamination with the Virus

All participants experienced high levels of fear of contamination with the virus. The majority reported that “dealing with the anonymous” makes our experience difficult as they might interact with asymptomatic people, while they do not know too much about this virus. All midwives declared that true fear had begun when the government started to enforce serious restrictions and mandated quarantine.

*“When I heard about the first case reported in Jordan and that the government closed the schools, universities, and all institutions, I had a panic attack, difficulty in breathing, difficulty in swallowing, and tremor … it was a difficult moment...we started working with the unknown thing”* (P10)

Many midwives experienced an unsubstantial feeling about the signs and symptoms of the disease and thought that they might become infected. For some, they had allergy in spring and each year they experienced similar symptoms, but with COVID-19 the experience was different. *“Every year I develop sore throat and headache in the spring season; however, this year, when I had similar symptoms, I was too scared and had to check my temperature more frequently, I could not sleep easily every day for one week….and I always thought that I might have the disease”* (P17)

This led to sleep disturbances, insomnia, and increased the stress level for some, while others had a panic attack. 

*“Oooh, at the beginning of this crisis (COVID-19 infection) my sleep was disturbed because I was thinking all the night of the possibility of carrying the virus and I do not know…you know, I see and have contact with many patients every day…how I can know…it’s an unknown thing; this is the problem”* (P6)

The level of fear reached the maximum among the participants when they looked after a suspected woman who had a COVID-19 test and was waiting for the result or when they cared for a woman with flue signs and symptoms like a cough. 

*“to be honest, I had more anxiety when I noticed flue sings in the admitted woman like cough or fever”* (P9) 

#### 1.b. Fear on Beloved Ones and Clients 

All midwives identified increased levels of stress and fear because they might have spread the virus to their beloved families and clients. As a result of their work nature and communication from hospital to their homes, while dealing with suspected clients, their levels of stress and fear increased. 

*“My fear from this virus increases at home ...I was scared that my children might get infected more than me …. I felt they were in danger …especially my little baby”* (P16)

Those living with elderly parents, and infant or very young children were the most worried, especially, breast feeding mothers. For some midwives, especially, those working with people who might be infected but not showing any signs and symptoms felt that they were “reservoir” of the virus and that they could spread the infection unintentionally, as seen in the following statements:

*“I live with my old parents and this makes me much worried when I go back home… I stay for a long time as much as I can in my private room…when I arrive home, I have a shower, and then greet them from a distance”* (P12)

#### 1.c. Being Physically Tired from High Workload 

Administrative midwives explained that based on the Jordanian Ministry of Health instructions to minimize the number of health workers, the administrative team prepared a new schedule to work with less than 50% of the personnel. This new arrangement increased the number of days off for midwives and kept them away from the hospital as much as they could. However, all midwives complained about the extra workload and the shortage of staff. A midwife working at the emergency department said: 

*“We are so tired…On the first day of the quarantine, the clients’ number reached 140 cases… we were exhausted…we could not take a break during our shifts”* (P14)

Midwives reported that due to the closure of the antenatal clinics and private clinics, all cases of low and high risk referred to the hospital in massive numbers.

*“It’s good to take extra days off as per new protocol, but it is too much work…all cases now come here, pregnant, vaginitis, UTI, scan …we are dying at work… we go back home very tired”* (P9)

#### 1.d. Being Uncomfortable from Extra Precautions 

At the beginning of the new experience of COVID-19, many midwives experienced different levels of discomfort from wearing Personal Protection Equipment (PPE). They considered it a change in their routine and work environment and the compliance with precautions decreased when there was no direct contact with clients for different reasons. Midwives reported feeling hot from wearing the gown for a long time. Some midwives complained of using the mask for a long time hours (16) during their shift and inhaling the same breath which leads to cough or allergy.

*“The main change in our work routine is wearing a gown, mask, gloves, and head and shoe covers all the time …I know we have to follow all instructions…but I have given up…the hospital is warm and wearing the gown, mask, and gloves makes it warmer and I have difficulty breathing”* (P1)

A midwife declared that wearing gloves all the time affected the performance of some skills and she felt uncomfortable. Some midwives also complained of hand skin dryness due to frequent hand washing. Besides, some midwives criticized the quality of the mask and described it as useless.

*“I have severe skin dryness due to the excessive hand washing and sterilizers… its quite annoying”* (P4)

### 2. Communication Dilemma 

#### 2.a. Midwife-client Communication

All midwives stressed on women’s right in receiving the appropriate care regardless of the risk of being contaminated. The majority of midwives declared that the care provided to clients did not change during the COVID-19 crisis, but with more precautions. 

*“This disease is not women’s mistake, so they should receive the best care...my work and interaction are still as before the crisis but while wearing a gown, gloves and a mask”* (P15)

Some midwives, however, reported some alterations in the physical interaction with women, for example, avoiding close distance, especially to the face, and minimized contraction assessment and touch. Some midwives criticized their colleagues when they received a suspected case in the emergency department as follows:

*“I tried to provide the best care for my client, but in this crisis, I avoid to undertake some assessments like contraction assessment and observe the CTG instead”* (P16)

#### 2.b. Midwife-colleague Communication

The majority of midwives reported that social greeting practices like shaking hands, kissing, and hugs were stopped and replaced by saying hello only. They also highlighted the changes in sharing food at breakfast or coffee breaks as many midwives bring their snacks from home although they eat at the same time. 

*“Before the crisis, we used to have breakfast and coffee together, but now, I bring my sandwich and water bottle from my home…we do not prepare coffee at work now”* (P7)

Some midwives reported that social distancing was maintained by many colleagues and social chat was minimized during this crisis. Another one expressed her anger from a colleague who was uncompliant with precautions and refused to share any food with her. 

*“No hugs, no kissing or hand shaking during Covid 19….my colleagues now keep the social distance as they can; we minimize the social chat and talk mainly about work issues”* (P11)

On the other hand, some midwives reported usual interaction and chatting time with their colleagues and having coffee breaks together, especially, with close friends and considered that as a kind of support in this situation as seen in the following statement:

*“I cannot change my greeting to my close friend….no change in our communication…. I have my breakfast and coffee break as usual…God might bless us”* (P3)

### 3. Positive Experience Over Time

#### 3.a. Becoming Less Overwhelmed 

For many midwives, the fear level was at its maximum during the first week of the crisis and decreased with time, especially when the number of infected people was decreasing. 

*“Thanks God, the situation is now better… now we accept the way of work and the level of fear has decreased; it is not like the first week of the crisis… the number of infected people is less”* (P1)

Midwives reported that the routine precautions and the new work environment became the norm. Despite the discomfort highlighted by midwives while using PPE in the first week, most midwives strictly followed all available types of PPE including frequently wearing gowns, gloves, masks, head and shoe cover, and frequent hand washing. 

*“Daily work and precautions have become a routine…sure wearing these precautions makes me work in a more relaxed way”* (P5)

These midwives emphasized the importance of following infection control instructions. They also considered the PPE as the main method to work with less stress and fear. Thus, they accepted wearing PPEs better than the previous period, as captured in the following quote:

*“Before the crisis, I used to wear gloves only when examining the clients or performing invasive procedures…now everything is different…now I do not remove the gloves for 16 hours sometimes to protect myself and others…by wearing gloves and masks, I can provide the care as usual”* (P18) 

#### 3.b. Knowledge Enhancement 

For some, gaining more information about the disease in general and about the protection methods decreased the level of stress. Some midwives searched for information personally. For example, two midwives took an online workshop about COVID-19 in general and had a certificate. Some midwives searched the Internet, gained general information, and felt themselves better when they knew more about the virus and protection methods. 

*“Everything is a routine now… we know more about this disease in general …however, we still take care to protect ourselves and others and need more information…but the fear now is less…the fear level sometimes decreases to 3 out of 10”* (P16)

### 4. Seeking Support

#### 4.a. Need to Access Information Resources 

All midwives recognized that they had a lack of knowledge about the COVID-19 effect on maternal and newborn health. All of them knew about the disease generally like the signs and symptoms and the precautions. They complained about the lack of information resources as the organization or the Ministry of Health did not offer courses or leaflets about the relationship between the disease and maternal health. 

*“I have my information like all people from TV, Ministry of Health (MOH) daily report, WhatsApp, and Facebook….to be honest, the MOH sent pamphlets and flyers about the disease signs and symptoms, transmission ways and precautions steps…. what we needed is a valid written material about COVID-19 and its effect on maternal and newborns to answer the mothers’ questions”*(P2)

All midwives gained their knowledge from social media, TV, and Internet webpages. However, their need to access valid and reliable scientific resources from their managers and administrators were clear during the interviews. They justified this need by insufficient time for them to access reliable resources either at work or at home.

*“Really, I do not have enough knowledge about COVID-19 and maternity care in specific. What I know is the disease sign and symptoms and the precautions needed… I cannot answer any questions related to pregnant woman’s health and her baby…I don’t have time to access scientific journals as I have workload here (hospital) and you know the heavy tasks at home with my kids”* (P20)

#### 4.b. Need to Improve the Work Environment 

All midwives were overwhelmed by the workload and suggested solutions to resolve this problem. Despite the vision of the administration in decreasing the number of staff, midwives needed more staff on each shift, even one more midwife on the shift. 

*“We are tired…please, we need more colleagues on the shift…three midwives on a 16-hour shift is not enough to take care of 80 to 100 cases daily”* (P9)

The second suggestion was to assign a midwife or a doctor with the civil defense team as a triage person to bring the most priority cases to the hospital.

*“I suggest one midwife or doctor should be with the civil defense team to sort out the cases and bring the most priority cases…the civil defense team brings all cases because they do not have enough knowledge”* (P14)

Midwives were unsatisfied with some points of management plans that were undertaken during the crisis. First, the cut from their annual leave: as the extra days off was provided to midwives to keep them away from the hospital environment, they were deducted from their annual holidays. 

*“When I saw my schedule, I was happy with the extra days off …but then I was surprised when I knew that all of these offs will be deducted from my annual vacation…then, when I finish them they will start to reduce my salary…. I have financial problems…you know I need my annual leave”* (P3)

Also, they expected more appreciation and rewards from the Ministry of Health. Therefore, midwives felt being disappointed; one of them said:

*“Thanks for the MOH for their efforts in protecting us and appreciating us as a frontline on the media …we were visited by a group from the Nursing and Midwifery’ Council to thank us for our job; however, we thought that they will provide us with rewards because all other employees stayed at home and we were here fighting this crisis”* (P16)

## DISCUSSION

Up to the author’s knowledge, this is the first Jordanian study that explored the midwives’ experiences during the difficult time of the COVID-19 pandemic crisis. In the same line with previous studies, ^5 , 18 - 20^ Jordanian midwives experienced a very tough time with incredible stress and fear from being infected or transmitting this virus, in particular, to their loved ones such as their children and old parents. This is supported by a previous study that reporting that the main source of stress and fear among HCPs is taking the virus home. ^21^ This makes some midwives feel more stressed at home more than when they are at the hospital, especially at the beginning of the crisis as reported in another qualitative study. ^6^ Midwives in the current study worked under pressure and had fluctuating emotions since the beginning of the crisis that affected their usual life such as sleep disturbance. Researchers also found that HCPs had lower sleep quality than other professionals and normal people. They also linked this poor quality of sleep to stress and depressive symptoms among their samples. ^22 , 23^ Other midwives’ illusions about being affected by the virus after taking care of suspected cases or those who had similar signs or symptoms of the disease were also reported. This kind of fear was documented and can generate mental distress and worsen psychiatric symptoms. ^24^ Therefore, early assessment of the midwives’ psychological status is important during this crisis. Maintaining the mental health of HCPs is essential to control infectious diseases. ^25^

Like other countries, the Jordanian government enforced national measures to minimize the spread of the COVID-19 disease. Thus, closing all health centers, public and private obstetric and gynecologic clinics, and working with less than half of the medical staff resulted in a massive increase in the number of clients. Consequently, midwives in the current study felt physically exhausted and fatigued due to the increasing midwife-client ratio and this is consistent with the findings of previous studies. ^5 , 6^ In Jordan, the shortage of midwives was reported before the crisis with an average ratio of midwives per person of 3.22 midwives per10000 population. ^8^ Therefore, the crisis increased the pressure on midwives when they received 100 to 140 clients on the same shift, as reported by the current study participants. Midwives suggested that a triage team should accompany the civil defense teams to sort out all cases before coming to the hospital to decrease this load. This suggestion was adopted in a hospital that implemented temporary strategies including online clinics and consultations, temporary separation of emergency cases, and cases that require routine check-ups to decrease the workload. ^26^ Midwives also requested their managers to increase the staff numbers again. Therefore, managers and leaders should develop different approaches to support HCPs and meet their needs during the crisis. ^21^

Many midwives at the beginning of the crisis experienced some discomfort while wearing PPE for a long time. Similar discomfort including sweat, headache, and difficulty in breathing, chest tightness, and palpitation were reported by HCPs in previous studies. ^5 , 6^ However, with time, the majority of our participants accepted the new routine and strictly followed all guidelines including wearing the PPE such as surgical masks, gowns, gloves, and hand washing to work in a safer and more relaxed environment. Providing midwives with good quality and enough PPE may contribute to their compliance with precautions and alleviate their stress. ^5^

Midwives in this study highlighted the client’s right in receiving the best care at any time and any circumstance. However, many midwives declared some alterations in interacting with their clients including decreased nonverbal communication while providing other essential care. Researchers recommended that HCPs could monitor the client from distance and provide care with minimal unnecessary contact. ^28^ Midwife-colleagues’ interaction was also altered in this study as many routines and daily practices were changed to minimize the risk of contamination and protect themselves and this is supported by a previous study. ^27^ However, the way of work and cooperation between them did not change. All participants stressed on respecting and appreciating all HCPs and the civil defense on their huge efforts during this crisis. Accordingly, midwives in the current study and previous studies recommended improving the work environment for midwives by reducing work intensity and pressure, giving more time for holidays, scientifically arranging the shifts, and financial rewards. ^29 - 31^ Therefore, such rewards and appreciation would be needed from managers and leaders to improve the psychological state of the midwives and help them to continue in the same manner of work under this pressure. ^17^

Moreover, midwives realized their lack of knowledge about the COVID-19 and maternal and newborn health and suggested providing them with updated knowledge from valid resources from the Ministry of Health, so that they can pass this information to their clients. Chinese researchers found that lack of knowledge about new infectious diseases at the first stage of the disease spread was a reason for negative emotions among HCPs. ^6^ Therefore, providing them with updated knowledge and training might decrease their fear and stress level. ^5^

As this is a qualitative study, generalizability of the results is limited to similar situations and cultures. Due to the quarantine and use of the snowball method, data were collected from two hospitals; hence, multicenter studies are recommended to be conducted. The years of experience of all participants were more than 10 years; including newly graduated midwives might reveal different themes. 

## CONCLUSION

Jordanian midwives experienced a very stressful and interruptive routine life while providing care to their clients during the COVID-19 outbreak. Many challenges, discomfort and uncertainty were revealed from this distinguished study. Midwives reported many needs from their managers and policymakers. This study recommends more attention to be paid to the midwives’ experiences and factors that might affect the quality of care. Thus, frequent assessment and discussions with midwives to identify their needs might be beneficial. The management and administrative team should do their best to reduce the work pressure and arrange the shifts and holidays appropriately. Policymakers and the government should provide the midwives with a safe work environment and updated information resources about the new infectious disease, in particular, about its effects on maternity and newborn care. Also, emotional and financial subsidies and rewards should be taken into consideration.
